# Native *N*-glycopeptide thioester synthesis through *N*→*S* acyl transfer

**DOI:** 10.1016/j.bmcl.2011.05.059

**Published:** 2011-09-01

**Authors:** Bhavesh Premdjee, Anna L. Adams, Derek Macmillan

**Affiliations:** Department of Chemistry, University College London, 20 Gordon Street, London WC1H 0AJ, UK

**Keywords:** Glycopeptides, Native chemical ligation, Peptide thioesters

## Abstract

Peptide thioesters are important tools for the total synthesis of proteins using native chemical ligation (NCL). Preparation of glycopeptide thioesters, that enable the assembly of homogeneously glycosylated proteins, is complicated by the perceived fragile nature of the sugar moiety. Herein, we demonstrate the compatibility of thioester formation via *N*→*S* acyl transfer with native *N*-glycopeptides and report observations that will aid in their preparation.

## Graphical abstract

We describe experiments that give us a sense of the impact of native chemical ligation, on *N*-glycopeptide thioester synthesis via *N*→*S* acyl transfer.

Glycoproteins are increasingly becoming viewed as tractable synthetic targets for development and exploitation as therapeutics.^1^ Consequently, efficient methods for the preparation of glycopeptide thioesters, the key building blocks for protein synthesis using native chemical ligation (NCL), are highly desirable.^2^

Previously, we showed that peptide thioesters (**1**) can be prepared via *N*→*S* acyl transfer from readily available precursors such as **2**, equipped simply with a C-terminal cysteine residue (Scheme 1).^3^

While demonstrating a preference for thioester formation at certain motifs over others,^3^ several additional factors appear to influence this transformation. In particular, variables such as peptide concentration, solubility and complexity appear important but have not been dissected in detail. Peptide thioesters adorned with chemically fragile post-translational modifications such as glycosylation are likely to benefit most from an optimized reaction protocol. Therefore model experiments were conducted with a view to accessing native *N*-glycopeptide thioesters that could be used for glycoprotein assembly.

A model *N*-glycopeptide (Scheme 2) corresponding to erythropoietin (EPO) residues 22–29 was prepared (see Supplementary data for experimental details). Acetate esters were retained on the sugar moiety to aid analysis and purification and prevent the fully deprotected glycopeptide from eluting too rapidly from the reverse-phase column. Glycopeptide **3** was subjected to thioester formation at 1 mg ml^−1^ (approx. 0.9 mM) peptide concentration in 0.1 M Na phosphate buffer; pH 5.8, containing 10% w/v sodium 2-mercaptoethanesulfonate (MESNa) and 0.5% w/v *tris*-carboxyethylphosphine (TCEP) at 55 °C for 48 h. The reaction was monitored by HPLC, and LC–MS, and showed smooth transition to thioester **4** within 48 h (Fig. 1a). Furthermore, ^1^H NMR spectroscopy of the purified product (Fig. 1b) confirmed thioester formation. Conversion to the desired thioester was additionally supported through NCL between **4** and an EPO fragment corresponding to residues 29–166 (Fig. 1c). The large EPO fragment was expressed in *Escherichia coli* and isolated as previously described.^4^ The ligation reaction was conducted in 0.3 M sodium phosphate buffer; pH 7.0 containing 6 M guanidine·HCl, 0.1 M 4-mercaptophenylacetic acid, and 40 mM TCEP. After 3 h LC–MS indicated that the initial recombinant fragment had been consumed. Treatment of the ligation reaction mixture with hydrazine hydrate in the presence of dithiothreitol for 1 h afforded the full length glycopolypeptide (Fig. 1d).

While the model experiments demonstrated that *N*-glycopolypeptides can be assembled using simpler *N*-glycopeptide thioesters formed through *N*→*S* acyl transfer, the isolated yield of **4** was rather low (20–40%). We considered thioester formation, as depicted in Scheme 1, to be essentially unidirectional, since the relatively low pH (pH 2–6) and high concentration of thiol additive (usually 10% w/v MESNa) employed are known to inhibit NCL.^5^ Consequently ligation between the thioester products and the released C-terminal cysteine should be negligible under the reaction conditions. However, the observation that thioester synthesis did not proceed to completion meant we could not rule out the possibility that competing NCL compromised reaction efficiency. If NCL was to influence the reaction outcome then we postulated that lowering the initial peptide concentration and reaction pH (from pH 5.8 to pH 2.0) should benefit thioester formation.

To test this hypothesis, a model peptide **5** (Sequence: H-MEELYKSHC-NH_2_) derived from the C-terminal residues of green fluorescent protein was first subjected to thioester formation in the presence of increasing concentrations of d-cysteine·HCl. We envisaged that overall epimerization of the C-terminal cysteine resulting from the cysteine exchange reaction (Scheme 3) should, in itself, produce products that were sufficiently resolvable so as to assess the occurrence of NCL by HPLC. Furthermore, addition of d-Cys-OH, rather than d-Cys-NH_2_ to the reaction mixture facilitated simpler detection of **7** by virtue of the C-terminal carboxyl, rather than carboxamide group present in **5**, which additionally allowed **5** and **7** to be unambiguously distinguished by mass.

In the absence of d-cysteine, thioester **6** was observed to accumulate to approximately 60% conversion after 48 h with hydrolysis to **8** appearing as the only significant side reaction (Fig. 2).

Surprisingly, **7** could be observed in the reaction mixture upon addition of less than 1 equiv of d-Cys and could accumulate to become a significant constituent of the reaction mixture at less than 10 equiv. As the concentration of d-cysteine increased (>350 equiv) in the reaction mixture thioester formation was dramatically reduced to only 30%. At high d-Cys concentrations an additional product, **9**, potentially corresponding to a disulfide-bonded dimer of **7** was also observed. Inter- or intramolecular disulfide bond formation is also a competing reaction that can inhibit *N*→*S* acyl transfer, although no disulfide-bonded adduct between **5** and d-Cys could be observed. Thioester formation in the presence of d-Cys was additionally investigated at lower pH (pH ∼2) employing 10% v/v AcOH as solvent (see Supplementary data). In this case, the reaction proceeded essentially as before although thioester hydrolysis appeared slightly reduced.

The results suggest that such reversibility in amide bond formation, enabled by an NCL/retro-NCL sequence, could be extended to dynamic combinatorial processes. However, it is unlikely that, under usual reaction conditions, the single equivalent of cysteine that is released will significantly compromise thioester production through NCL.

In light of these findings it was subsequently unsurprising that a fivefold reduction in the concentration of our glycopeptide thioester precursor **3** did not dramatically influence the production of **4**, as determined by HPLC. Notably, **4** appeared to accumulate more rapidly when the reaction was conducted at lower concentration. Furthermore, lowering the reaction pH, to inhibit NCL, did not give rise to an appreciable amount of **4** after 48 h. Instead a complex mixture of products resulting from extensive deacetylation of both **3** and **4** was obtained.

In summary, our results show that thioester formation via *N*→*S* acyl shift is compatible with native *N*-glycopeptides at pH 5.8. Deacetylated sugars should be employed in reactions conducted at lower pH otherwise partial sugar deacetylation can complicate analysis. These simply glycosylated thioesters can, through NCL, be assembled into glycoproteins.^6^ Furthermore, we investigated the potential for NCL to occur during thioester formation using a cysteine exchange reaction and found that NCL can clearly reverse thioester formation. However, this should not be significant under normal reaction conditions. The results also emphasize the remarkable dynamic nature of amide bonds susceptible to an NCL/retro-NCL process, even in the absence of synthetic ‘devices’^7^ or inteins.^8^

## Figures and Tables

**Figure 1:**
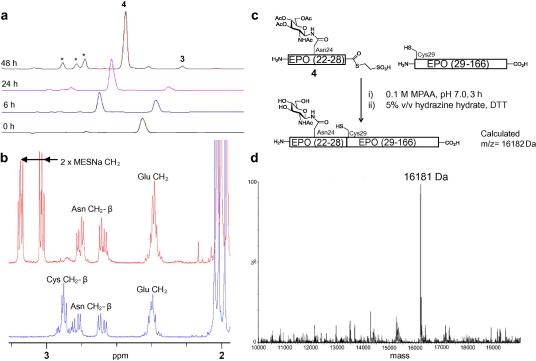
(a) HPLC analysis of thioester formation. Peaks marked with asterisks correspond to derivatives of **3** or **4** with a cleaved acetate ester. (b) ^1^H NMR analysis of the purified thioester **4** (upper trace) and **3** (lower trace, see Supplementary data for full spectrum). (c) NCL between **4** and recombinant EPO fragment comprising residues 29–166. (d) ESI mass spectrum of the crude ligation product following carbohydrate deacetylation.

**Figure 2:**
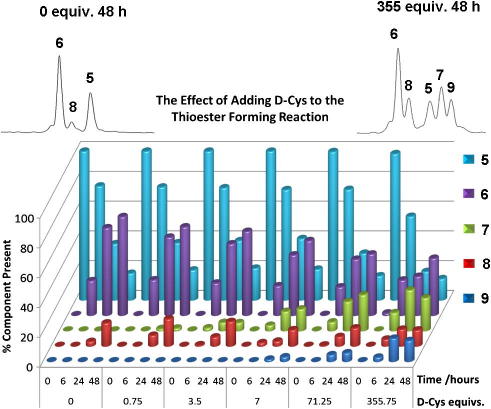
Relative composition (%) of the reaction mixture as a function of time and added d-Cys·HCl. The reactions were all conducted at 1 mg ml^−1^ (approx. 0.9 mM) peptide concentration in 0.1 M Na phosphate buffer; pH 5.8, 10% w/v MESNa, 0.5% w/v TCEP·HCl, 60 °C, 48 h.

**Scheme 1:**
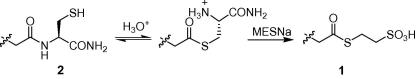
Peptide thioester synthesis employing an *N*→*S* acyl shift.

**Scheme 2:**
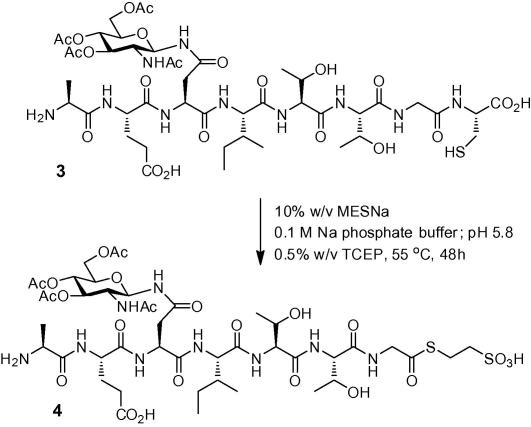
*N*-Glycopeptide thioester formation at pH 5.8.

**Scheme 3:**
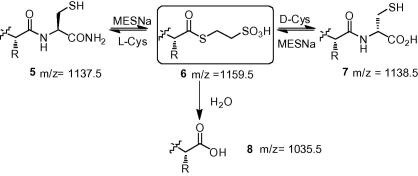
Thioester formation and competing NCL were investigated, under identical reaction conditions, by conducting the reaction in the presence of d-cysteine.
