# Supplementary material for: Native N-glycopeptide thioester synthesis through N→S acyl transfer
Source: Bioorg Med Chem Lett. 2011 Sep 1;21(17):4973–5. doi: 10.1016/j.bmcl.2011.05.059 (PMC3160546; doi:10.1016/j.bmcl.2011.05.059)

Supporting information: Native *N*-glycopeptide thioester formation through N→S acyl transfer

Bhavesh Premdjee, Anna L. Adams, and Derek Macmillan*

Department of Chemistry, University College London, 20 Gordon Street, London, WC1H 0AJ, UK.

Synthesis of glycopeptide thioester 4

General Methods

Chemicals were obtained from Novabiochem, Sigma-Aldrich and Fluka, and were used without further purification. Petroleum Spirits refers to petroleum ether that boils in the range 40-60°C. Flash chromatography was carried out with silica gel 40-63 µm (VWR). Thin layer chromatography (TLC) was performed on Merck Silica gel 60 F_{254} aluminium sheets (0.25mm layer coating) and was visualised with short wavelength (366nm) ultraviolet light. Staining was performed with a solution of anisaldehyde, using heat to visualise spots. Proton Nuclear Magnetic Resonance (^{1}H NMR) were recorded at 500MHz or 600 MHz on Bruker AMX500 and AMX600 instruments respectively. ^{13}C NMR experiments were recorded on the same instruments at 125MHz or 150MHz. All NMR experiments were recorded at room temperature. Chemical shifts (δ) are recorded in parts per million (ppm) and reported with reference to the solvent peak, with δ_H values reported to 2 decimal places and δ_C reported to 1 decimal place. $CDCl_3$ refers to fully deuterated chloroform and DMSO-d_6 refers to fully deuterated dimethyl sulfoxide. Signals are denoted as s = singlet, d = doublet, t = triplet, q = quartet, dd = doublet of doublets and m = multiplet. Coupling constants (*J*) are reported in Hertz and were calculated from observed chemical shifts and are given to 1 decimal place. FAB (fast atom bombardment) and ESI (electrospray ionisation) mass spectrometry experiments were performed by Dr Lisa Harris from the Department of Chemistry at UCL.

2-acetamido-2-deoxy-3, 4, 6-tri-*O*-acetyl-α-D-glucopyranosyl chloride (S2)

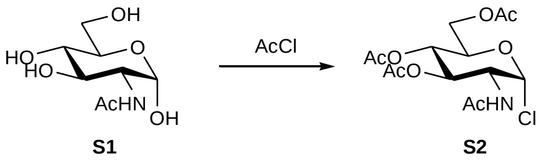

N-acetyl glucosamine **(S1)** (10.17 g, 46 mmol) was added to stirring acetyl chloride (20 mL) in a flask equipped with a drying tube. The suspension was stirred for 18 hours and formed an amber coloured solution, to which chloroform (80 mL) was added. The resulting solution was poured into ice (80 g) and water (20 mL) with stirring. The organic layer was run into saturated sodium

bicarbonate (80 mL) with neutralisation being completed in a separating funnel. The organic layer was then dried over anhydrous $MgSO_4$ for 15 minutes before being filtered with suction. The yellow solution was then evaporated to dryness *in vacuo* to afford the crude product as a light brown solid, which was subsequently purified by flash chromatography over silica (100% EtOAc) to give the product as an off white solid (5.96 g, 36%). ^{1}H NMR (500 MHz, $CDCl_3$) δ 6.18 (d, *J* = 3.7, H1), 5.83 (d, *J* = 8.5, NH), 5.32 (t, *J* = 10.4, H3), 5.21 (t, *J* = 9.5, H4), 4.51 - 4.55 (m, H2), 4.27 (m, $H6_a$ and H5), 4.13 (dd, *J* = 12.5, 2.0, $H6_b$), 2.10 (s, CH_3), 2.05 (s, 2 x CH_3), 1.98 (s, CH_3). ^{13}C NMR (125MHz, $CDCl_3$) δ 171.6, 170.7, 170.2, 169.2, 93.7, 71.0, 70.2, 67.0, 61.2, 53.6, 23.2, 20.8, 20.6, 20.5. FAB$^+$ MS (*m/z*) calculated for $C_{14}H_{20}NO_8Cl$ 365.09 found $[MNa]^+$ 388.08.

2-acetamido-2-deoxy-3, 4, 6-tri-*O*-acetyl-β-D-glucopyranosyl azide (S3)

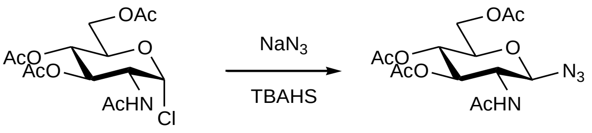

S2 **S3**

Glycosyl chloride **(S2)** (5.96 g, 16.3 mmol), tetrabutylammonium hydrogen sulphate (5.47 g, 16.3 mmol) and sodium azide (3.13 g, 48.1 mmol) in dichloromethane (50 mL) and saturated sodium bicarbonate (50 mL) was stirred vigorously at room temperature for 3 hours. To the biphasic mixture was added ethyl acetate (150 mL) and the organic layer was separated. This was washed with saturated sodium bicarbonate (100 mL) and then water (2 x 100 mL). It was then washed with saturated sodium chloride (50 mL) before being dried over $MgSO_4$. The organic mixture was then collected under vacuum and the volatile solvents were removed in a rotary evaporator. The pure product was precipitated from ethanol/petroleum spirit (3.58 g, 59%). ^{1}H NMR (500 MHz, $CDCl_3$) δ 5.73 (d, *J* = 8.8, NH), 5.24 (t, *J* = 9.6, H3), 5.09 (t, *J* = 9.6, H4), 4.75 (d, *J* = 9.3, H1), 4.26 (dd, *J* = 12.4, 4.8, $H6_a$), 4.16 (dd, *J* = 12.4, 2.0, $H6_b$), 3.92 (q, *J* = 9.2, H2), 3.78 (m, H5), 2.10 (s, CH_3), 2.04 (s, CH_3), 2.03 (s, CH_3), 1.97 (s, CH_3). ^{13}C NMR (125MHz, $CDCl_3$) δ 171.1, 170.8, 170.5, 169.4, 88.5, 74.1, 72.2, 68.1, 62.0, 54.2, 23.3, 20.8, 20.7, 20.6. ESI$^+$ MS (*m/z*) calculated for $C_{14}H_{20}N_4O_8$ 372.13 found $[MNa]^+$ 395.12.

2-acetamido-2-deoxy-3, 4, 6-tri-*O*-acetyl-β-D-glucopyranosyl amine (S4)

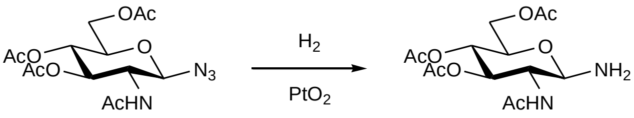

S3 **S4**

Glycosyl azide **(S3)** (2.01 g, 5.39 mmol) and platinum (IV) oxide (100 mg) were added to a 3 necked flask, which was evacuated and purged with nitrogen gas 3 times. Dry tetrahydrofuran (36 mL) was added and flask was evacuated and purged twice with hydrogen gas. The mixture was stirred under hydrogen at room temperature and monitored by thin layer chromatography. After 3 hours TLC indicated complete consumption of the azide and the reaction mixture was filtered through celite. The product was evaporated to dryness to yield the amine as a grey powder (1.41 g, 96%). ^{1}H NMR (500 MHz, $CDCl_3$) δ 5.66 (d, *J* = 8.8, NH), 5.01 - 5.13 (m, H3, H4), 4.21 (dd, *J* = 12.3, 4.9, $H6_a$), 4.09 - 4.12 (m, H1, $H6_b$), 4.02 (q, *J* = 9.4, H2), 3.63 (m, H5), 2.09 (s, CH_3), 2.04 (s, CH_3), 2.03 (s, CH_3), 1.99 (s, CH_3). ^{13}C NMR (125MHz, $CDCl_3$) δ 171.7, 170.9, 170.8, 169.4, 86.5, 73.5, 72.9, 68.5, 62.5, 55.0, 23.4, 20.9, 20.8, 20.7. ESI^+ MS (*m/z*) calculated for $C_{14}H_{22}N_2O_8$ 346.14 found $[MNa]^+$ 369.13.

N-α-fluorenylmethoxycarbonyl-N-β-(2-N-acetylamido-2-deoxy-β-D-glucopyranosyl)-2-tertbutoxy-L-asparagine (S5)

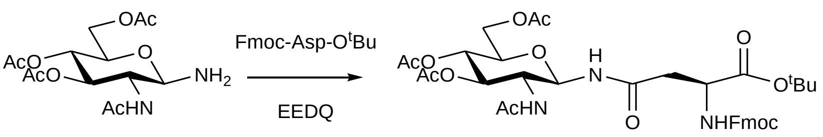

S4 **S5**

Glycosyl amine **(S4)** (500 mg, 1.44 mmol), Fmoc-Asp-O^tBu (594 mg, 1.44 mmol) and N-ethoxycarbonyl-2-ethoxy-1, 2-dihydroquinoline (357 mg, 1.44 mmol) were stirred in dichloromethane (45 mL) under nitrogen at room temperature for 4 hours. The crude mixture was concentrated *in vacuo* to afford a grey solid to which a mixture (1:1) of hot ethyl acetate and petroleum sprit (20 mL) was added. The suspension was filtered and the product was collected as a grey solid which was dried under vacuum to yield 821 mg (77%). ^{1}H NMR (500 MHz, $CDCl_3$) δ 7.75 (d, *J* = 7.5, 2 x CH), 7.59 (d, *J* = 7.4, 2 x CH), 7.39 (t, *J* = 7.3, 2 x CH), 7.30 (m, 2 x CH, NH), 6.21 (d, *J* = 8.3, NH), 5.96 (d, *J* = 8.6, NH), 5.03 - 5.11 (m, H1, H3, H4), 4.49 - 4.55 (m, $CH_α$), 4.41 (m, CH), 4.17 - 4.30 (m, 2 x CH, H6), 4.12 - 4.14 (m, CH, H2), 3.74 (dd, *J* = 9.7, 2.0, H5), 2.84 (dd, *J* = 16.4, 4.2, $H_β$), 2.70 (dd, *J* = 16.3, 3.9, $H_β$), 2.06 (s, CH_3), 2.05 (s, CH_3), 2.04 (s, CH_3), 1.94 (s, CH_3), 1.44 (s, $C(CH_3)_3$). ^{13}C NMR (125MHz, $CDCl_3$) δ 172.4, 172.0, 171.2, 170.8, 170.1, 169.4, 156.2, 144.0, 141.3, 127.8, 127.2, 125.2, 120.0, 82.3, 80.2, 73.6, 73.0, 67.7, 67.2, 61.8, 60.5, 53.4, 51.1, 47.2, 38.1, 28.0, 23.1, 20.8, 20.7, 20.6, 14.3. FAB^+ MS (*m/z*) calculated for $C_{37}H_{45}N_3O_{13}$ 739.30 found $[MNa]^+$ 762.29.

***N*-α-fluorenylmethoxycarbonyl-*N*-β-(2-*N*-acetylamido-2-deoxy-β-D-glucopyranosyl)-L-asparagine (S6)**

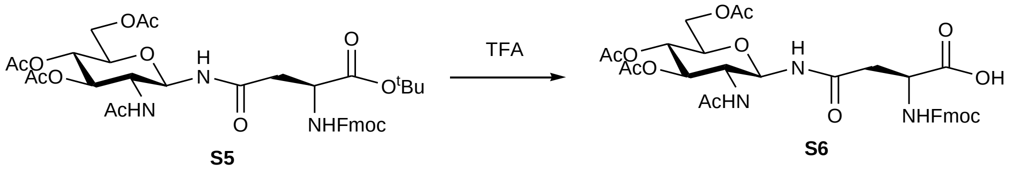

Glycoamino acid **(S5)** (500 mg, 0.68 mmol) was treated with 95% aqueous trifluoroacetic acid (10 mL) and stirred at room temperature for 3 hours. Thin layer chromatography analysis indicated completion of the reaction and the solvent was removed *in vacuo* to yield the product as a grey solid (462 mg, 100%). ^{1}H NMR (500 MHz, DMSO-d$_6$) δ 8.57 (d, *J* = 9.2, NH), 7.87 (d, *J* = 8.0, 2 x CH, NH), 7.69 (d, *J* = 7.4, 2 x CH), 7.45 (d, *J* = 11.5, NH), 7.40 (t, *J* = 7.4, 2 x CH), 7.31 (t, *J* = 7.4, 2 x CH), 5.17 (t, *J* = 9.9, H1), 5.08 (t, *J* = 9.9, H3), 4.80 (t, *J* = 9.8, H4), 4.38 (q, *J* = 8.7, CH$_\alpha$), 4.15 - 4.30 (m, H6$_a$, CH, CH$_2$), 3.93 (dd, *J* = 10.7, 1.6, H6$_b$), 3.86 (q, *J* = 9.7, H2), 3.80 (dd, *J* = 9.9, 1.5, H5), 2.64 (dd, *J* = 16.3, 5.4, CH$_\beta$), 2.48 (dd, overlapped by DMSO signal CH$_\beta$), 1.98 (s, CH$_3$), 1.95 (s, CH$_3$), 1.89 (s, CH$_3$), 1.70 (s, CH$_3$). ^{13}C NMR (125MHz, DMSO-d$_6$) δ 173.0, 171.5, 170.0, 169.8, 169.5, 169.3, 155.8, 143.8, 140.7, 127.6, 127.1, 125.2, 120.1, 78.1, 73.4, 72.3, 68.4, 65.7, 52.1, 50.0, 46.6, 36.8, 22.6, 20.5, 20.4, 20.3. ESI$^+$ MS (*m/z*) calculated for $C_{33}H_{37}N_3O_{13}$ 683.23 found [MNa]$^+$ 706.22.

***N*-linked glycopeptide synthesis of H-AEN((OAc)$_3$(GlcNAc)ITTGC-OH, 3**

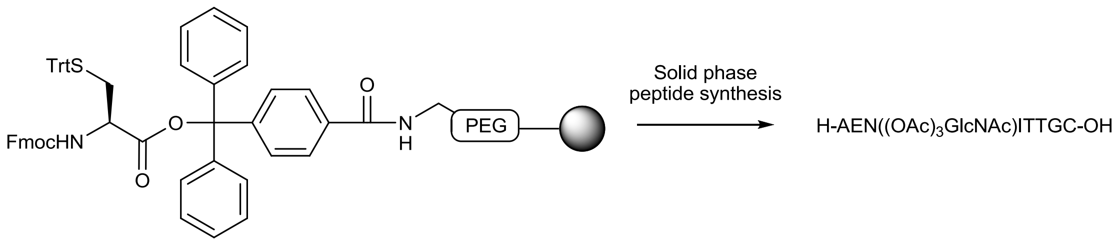

Fmoc-Cys(Trt)-NovaSyn®TGT resin (0.2 mmolg^{-1} loading) (0.5 g, 0.1 mmol) was deprotected with 20% piperidine in *N*, *N*-dimethylformamide (v/v) (3.0 mL) for 15 minutes. The resin was then washed with *N*, *N*-dimethylformamide (3 x 4.0 mL) followed by dichloromethane (2 x 4.0 mL). To the dry resin was added a stirred solution of glycine (297.3 mg, 1 mmol), 0.45 M 1-Hydroxybenzotriazole/ 2-(1H-benzotriazole-1-yl)-1,1,3,3-tetramethyluronium hexafluorophosphate (HOBt/HBTU) coupling reagent (2.2 mL), and di-isopropylethylamine (300 µL) in *N*, *N*-dimethylformamide (2.2 mL). The reaction mixture was shaken at 430 rpm at room temperature for 4 hours. The remaining amino acids were coupled in the same fashion using 1 mmol of Fmoc-Xaa-OH (10 equivalents). An exception to this was the glycoamino acid **(S6)** for which only 5 equivalents were employed. The peptide was cleaved by treating half of the peptide bound resin with a mixture of trifluoroacetic acid, ethanedithiol and water (95:2.5:2.5) for 5 hours. The mixture was filtered and washed with neat TFA (1.0 mL) and

crude peptide was precipitated from the filtrate with diethyl ether (4 x 8.0 mL) in 15mL Falcon tubes. The samples were centrifuged at 3000 rpm for 15 minutes and the supernatant was decanted off and discarded. The process was repeated again and the crude peptide was left to dry in a fume hood for an hour. Semi-preparative reverse phase HPLC was used to purify the peptide (gradient: 5 to 60% acetonitrile in water over 45 minutes). The peptide was found to have a retention time of 23 minutes and the collections were lyophilised to afford H-AEN($(OAc)_3$(GlcNAc)ITTGC-OH as a fluffy white solid (30.2 mg, 53% yield based on initial resin loading). ESI^+ LCMS (*m/z*) calculated for $C_{45}H_{72}N_{10}O_{22}S$ 1136.45 found $[MH]^+$ 1137.25.

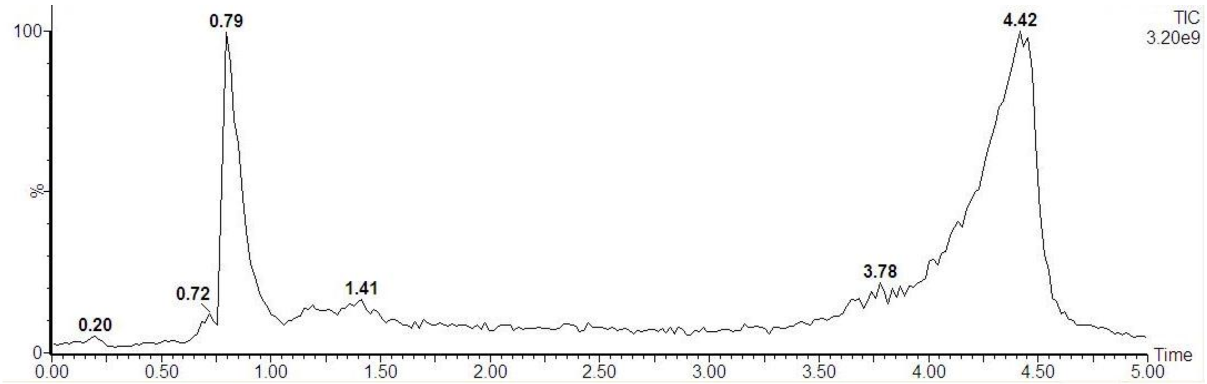

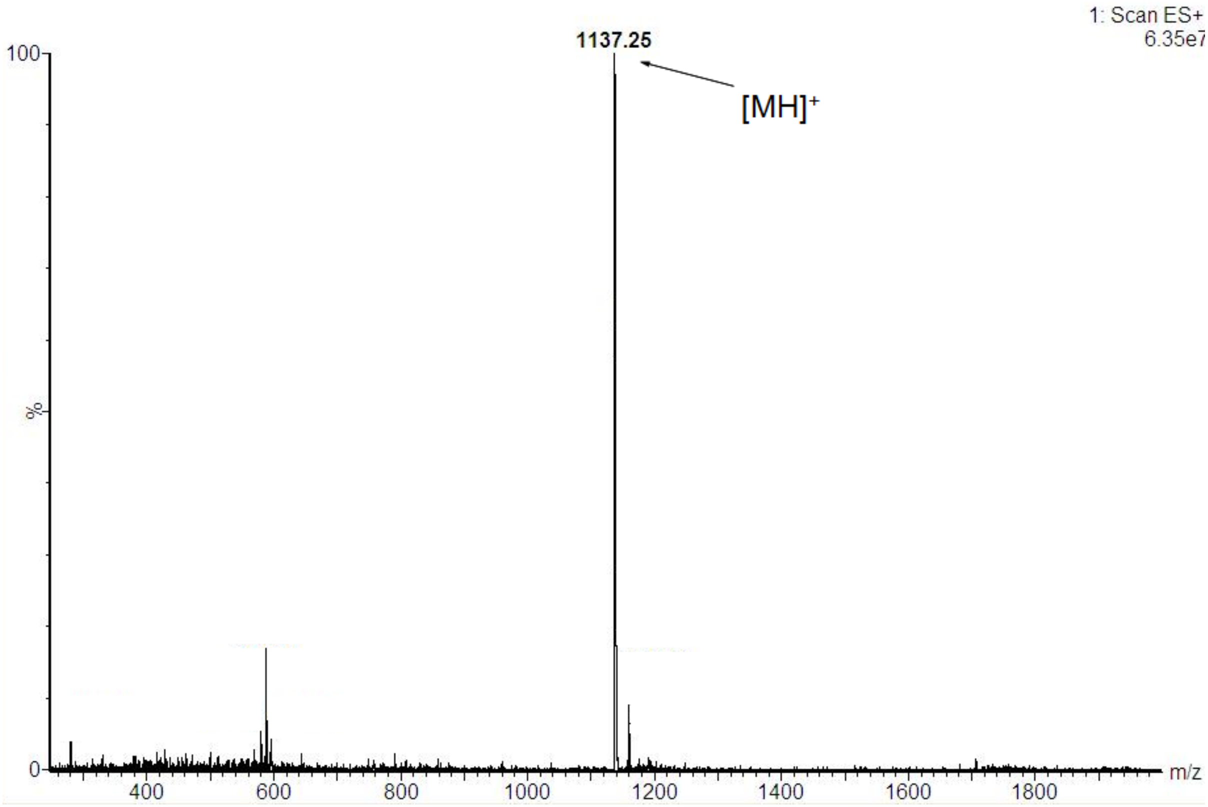

Synthesis of glycopeptide thioester of H-AEN((OAc)$_3$(GlcNAc)ITTG-SCH$_2$CH$_2$SO$_3$H

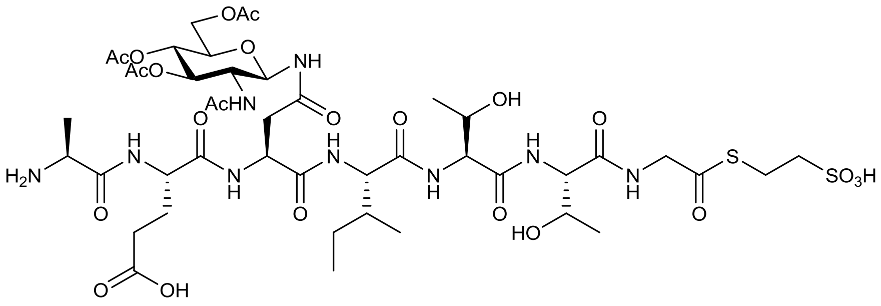

H-AEN((OAc)$_3$(GlcNAc)ITTG-SCH$_2$CH$_2$SO$_3$H

In 5 microcentrifuge tubes (1.5 mL) was added H-AEN((OAc)$_3$(GlcNAc)ITTGC-OH (to a final concentration of 1 mg/mL), sodium 2-mercaptoethanesulfonate (to a final concentration of 10% v/v), tris (2-carboxyethyl) phosphine hydrochloride (to a final concentration of 0.5% v/v) and sodium phosphate buffer (0.1 M at pH 5.8). The samples were agitated on an Eppendorf® Thermomixer at 55 °C and 500 rpm for 48 hours. The reaction was monitored by analytical reverse-phase HPLC (gradient: 5 to 95% acetonitrile in water over 45 minutes) at set intervals of 0, 6, 24 and 48 hours.

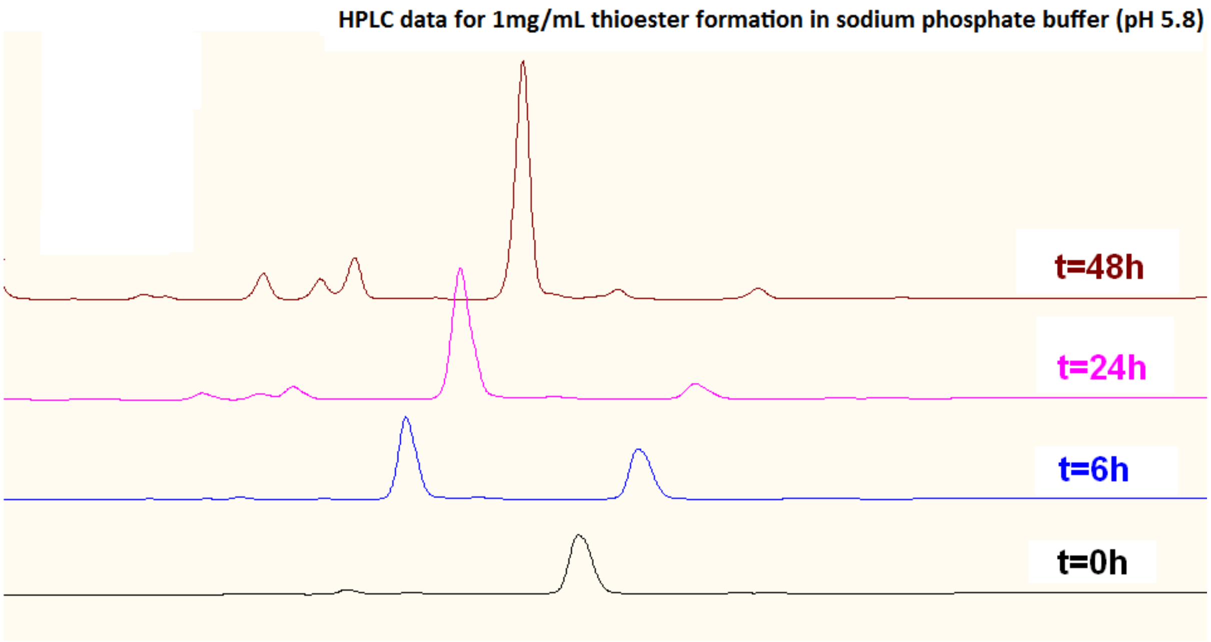

The thioester was purified directly from the reaction mixture by semi-preparative reverse phase HPLC (gradient: 5 to 60% acetonitrile in water over 45 minutes). The glycopeptide thioester was found to have a retention time of 22 minutes and the collections were lyophilised to yield the product as a fluffy white solid (1 mg, 21%). ESI$^+$ LCMS (*m/z*) calculated for $C_{44}H_{71}N_9O_{23}S_2$ 1157.41 found $[MH]^+$ 1158.3 and $[MNa]^+$ 1180.4.

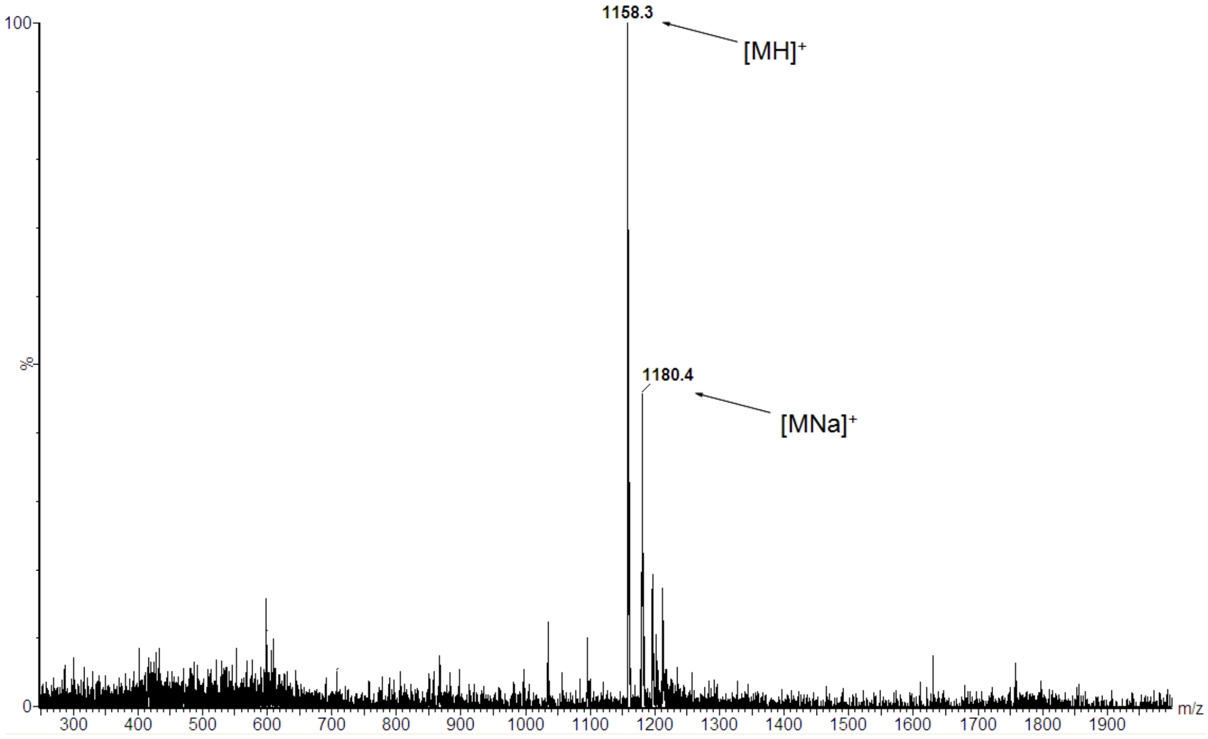

The reaction was repeated as described above using a peptide concentration of 0.2mg/mL (5 mg in 25 mL) in a round bottomed flask, heated on an oil bath. The reaction was also repeated using 0.2 mg/mL of peptide with 10% acetic acid instead of sodium phosphate buffer to give a pH ≈ 2.

HPLC data for 0.2mg/mL thioester formation in sodium phosphate buffer (pH 5.8)

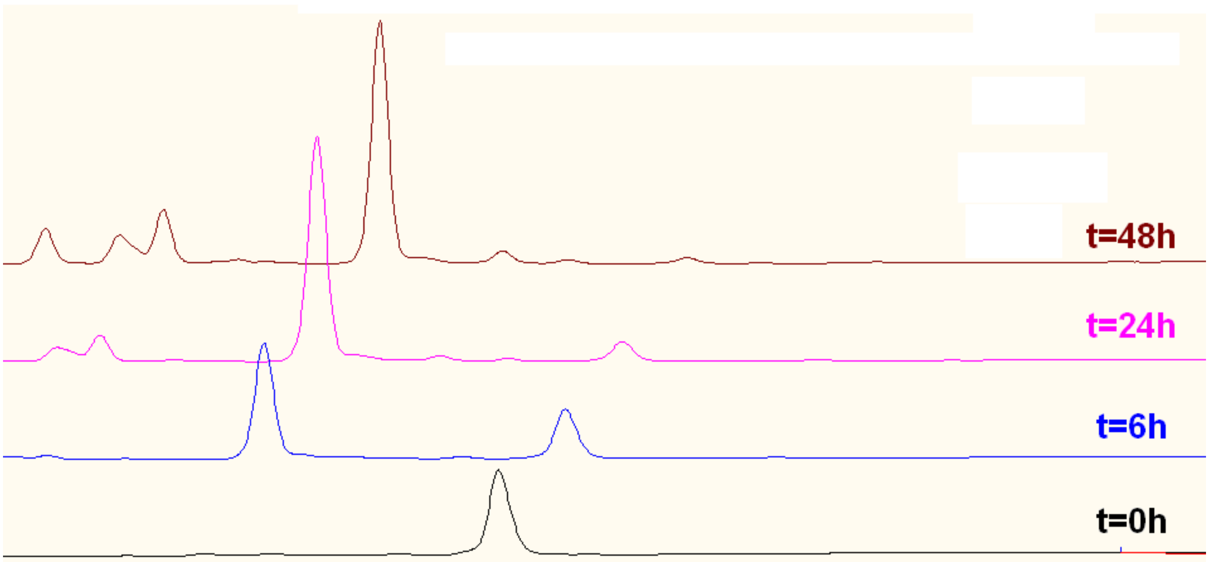

HPLC data for 0.2mg/mL thioester formation in 10% acetic acid (pH ≈ 2)

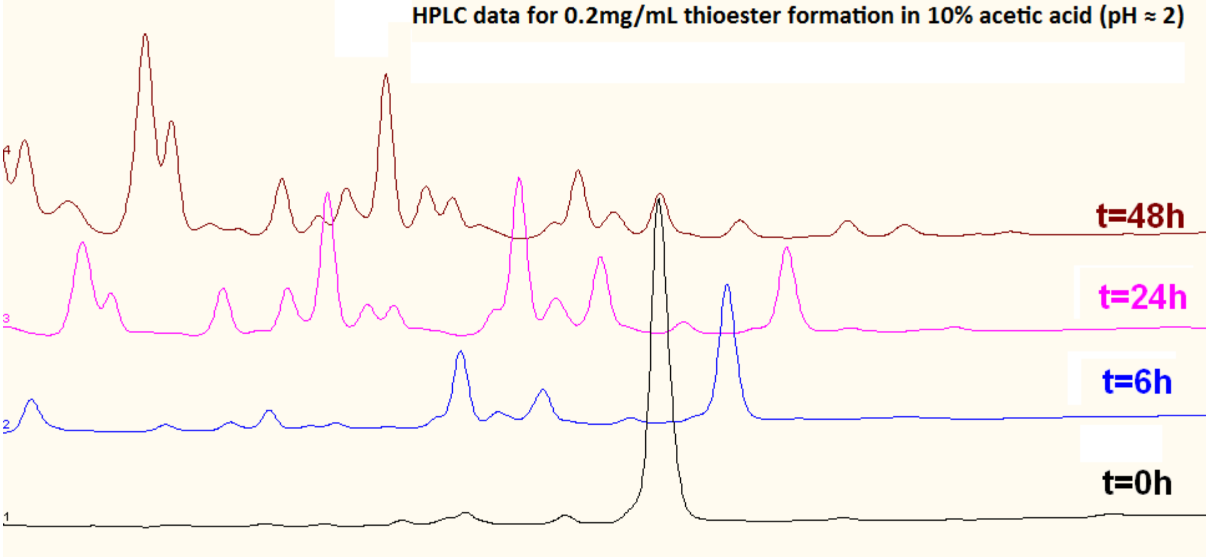

HPLC data for thioester formation at various conditions at 48h

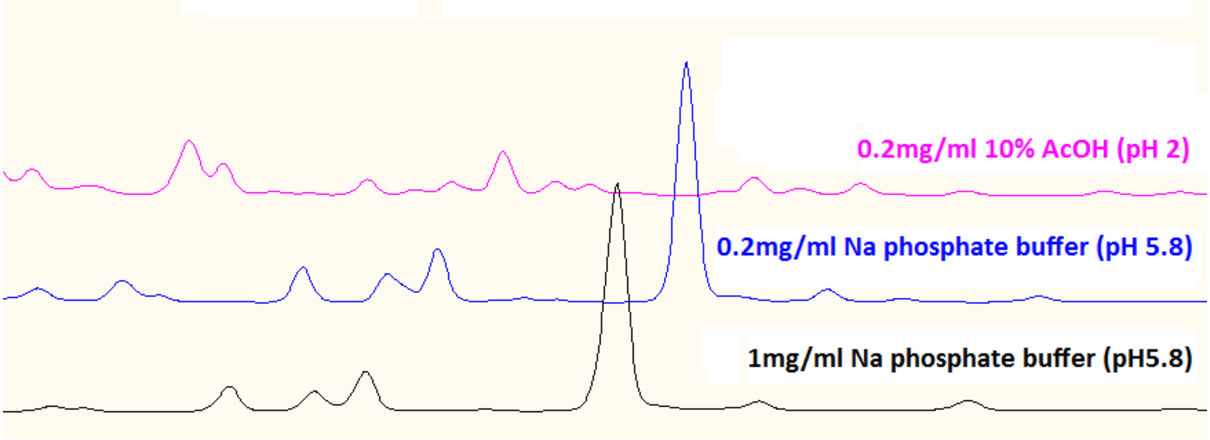

1H NMR spectra of **3** (lower trace) and **4** (upper trace)

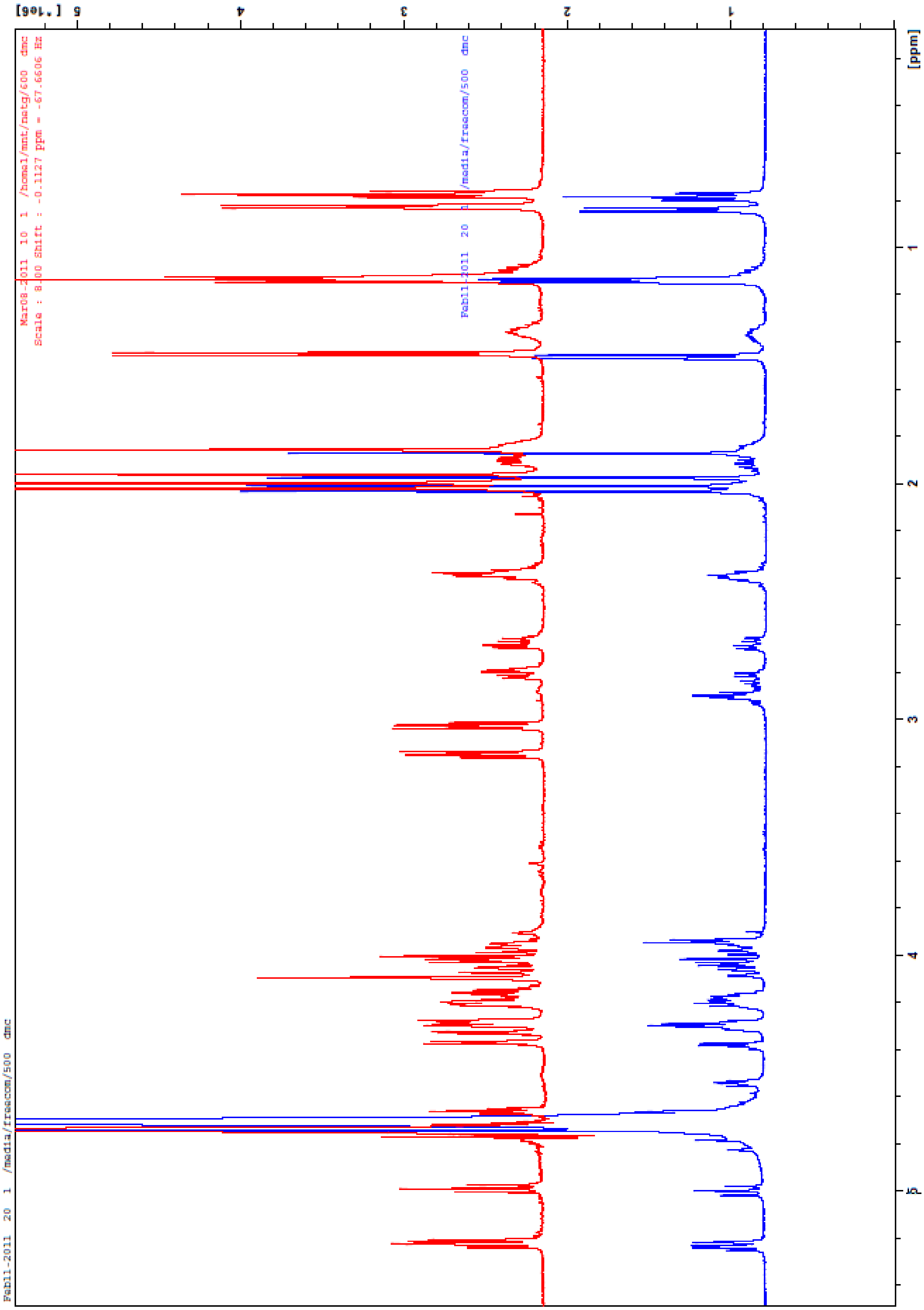

Native chemical ligation for the synthesis of EPO residues 22-166

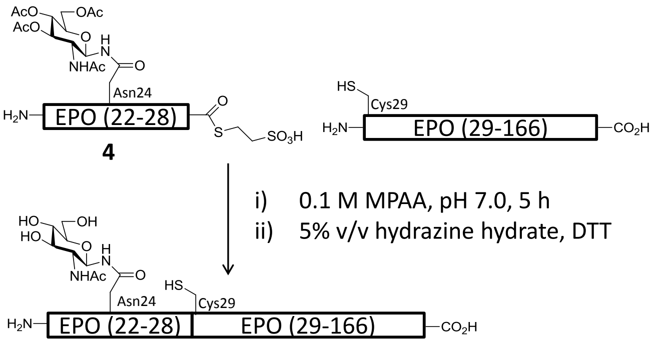

EPO 29-166 (2.5 mg) was dissolved in 6M Guanidine hydrochloride containing 0.3 M sodium phosphate buffer (pH 5.8, 0.5 mL). To the protein solution was added 20 µL of 1 M TCEP (neutralised to pH 7 with NaOH) and 50 µL of 1 M MPAA (neutralised to pH 7 with NaOH) to give final concentrations of 40 mM and 100 mM respectively. The resulting mixture was added to glycopeptide thioester H-AEN($(OAc)_3$(GlcNAc)ITTG-$SCH_2CH_2SO_3H$ (2 mg) in a microcentrifuge tube and shaken at 500 rpm under N_2 at room temperature on an Eppendorf Thermomixer. After 3 hours, LCMS indicated consumption of the protein EPO 29-166, and formation of the ligation product. The reaction mixture was subsequently diluted by the addition of cold distilled water (8 mL) and stored at 4 °C overnight. A white precipitate formed and the sample was centrifuged at 3000 rpm for 10 minutes. The process was repeated, resuspending the protein in cold distilled water (8 mL) and allowed to precipitate at 4 °C overnight. The crude protein was then collected by centrifugation and then treated with hydrazine hydrate (5% v/v) and DTT (50 mM) in 6 M guanidine hydrochloride (3 mL) at room temperature with gentle agitation. After an hour, LCMS indicated complete deacetylation of the starting material and the protein was isolated through dilution with water and precipitation as illustrated above.

Model Peptide Synthesis

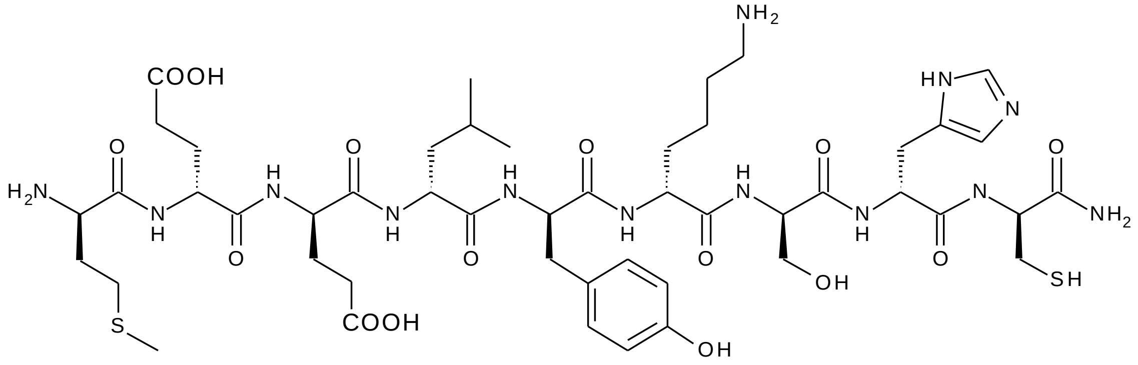

The model peptide, was derived from the C-terminal sequence of green fluorescent protein (GFP) and was synthesised on Rink Amide resin to afford the peptide carboxamide.

GFP-HC-NH$_2$ 5: Rink Amide-MBHA resin, loading = 0.56 mmol g^{-1}, (89.3 mg, 0.05 mmol) was treated with 20% piperidine in DMF (v/v) (1.0 mL, 15 min) to remove the Fmoc protecting group. The resin was then washed exhaustively with DMF then DCM. Fmoc-Cys(Trt)-OH (0.25 mmol) was then coupled to the resin using 0.45 M HBTU/HOBt (0.55 mL), DIPEA (75 µL) in DMF (0.55 mL), with shaking at room temperature for 4 hours. The resin was then washed and transferred to the automated peptide synthesiser (ABI 433A) reaction vessel for peptide chain elongation employing 10 equivalents of Fmoc-Xaa-OH for each residue up to the final methionine at the N-terminus. The resin was then removed from the synthesiser vessel and washed. The resin was treated with trifluoroacetic acid:ethanedithiol:water (95:2.5:2.5 = 4.0 mL) for 4 hours at room temperature, after which the resin beads were filtered off and the remaining solution was transferred into cold diethyl ether (10 x volume) to induce precipitation. The precipitate was collected by centrifugation (3000 rpm, 15 min) and the supernatant was decanted. The pellet was then washed with diethyl ether (10 mL) and centrifuged again (3000 rpm, 15 min). The supernatant was again removed and the pellet was dissolved in distilled water with acetonitrile (up to 40% as required) and purified by semi-preparative HPLC. The fractions containing the desired peptides (determined by LC-MS) were collected and lyophilised to afford a white solid peptide. **H-MEELYKSHC-NH$_2$:** Isolated by HPLC, t_r = 19-20 min as a fluffy white solid (16.6 mg, 30%). Calculated mass, 1138.31 Da $[MH]^+$; observed mass, 1138.45 Da $[MH]^+$.

The following reactions were followed by analytical HPLC using the Dionex UltiMate 3000 with a Phenomenex Sphere Clone (5 µm particle size 250 x 4.60 mm) column with a gradient of acetonitrile (containing 0.1% TFA) in water (containing 0.1% TFA) of 5%-95% over 45 minutes and a flow rate of 1 mL/min.

Thioester Formation in the Presence of Free D-Cysteine at pH 5.8

tGFP-H-$SCH_2CH_2SO_3H$: In six separate vessels, **5** (0.5 mg, 0.44 μmol) was dissolved in a solution containing 10 % (v/v) sodium phosphate buffer (50 μL, 0.1M, pH 5.8), 10 % (w/v) sodium 2-mercaptoethanesulfonate (50 mg) and 0.5 % *tris*(2-carboxyethyl)phosphine (2.5 mg, 9.99 μmol) in water (total volume 0.5 mL). To each vessel a different amount of D-cysteine hydrochloride was added (0 eq; 0.75 eq, 0.05 mg; 3.5 eq, 0.25 mg; 7.5 eq, 0.5 mg; 10 eq, 5 mg; 50 eq, 25 mg). The resulting mixtures were agitated at 60°C for 48 h in an Eppendorf thermomixer. Analytical HPLC revealed the presence of 2 new species forming during the reaction as a result of adding D-cysteine at 16.4 min and 16.7 min. These formed alongside the previously known thioester product (15.3 min), hydrolysed peptide, tGFP-H-OH (15.5 min) and starting material, tGFP-HC-NH_2 (16.1 min). Electrospray mass spectrometry (carried out on a Waters Aquity UPLC-SQD MS system with an applied voltage of 60 V) confirmed that these new components contained the epimeric peptide **7** (tGFP-H$\mathbf{C_D}$-OH) after 6 hours in the reactions with more than 0.75 eq D-cysteine hydrochloride. This was observed with 1 mass unit difference from the starting material due to the C-terminal comprising a carboxylic acid in place of the carboxamide of the starting peptide, calculated mass, 1139.31 Da $[MH]^+$; observed mass, 1139.63 Da $[MH]^+$.

Data for Thioester Formation in the Presence of Free D-Cysteine at pH 2

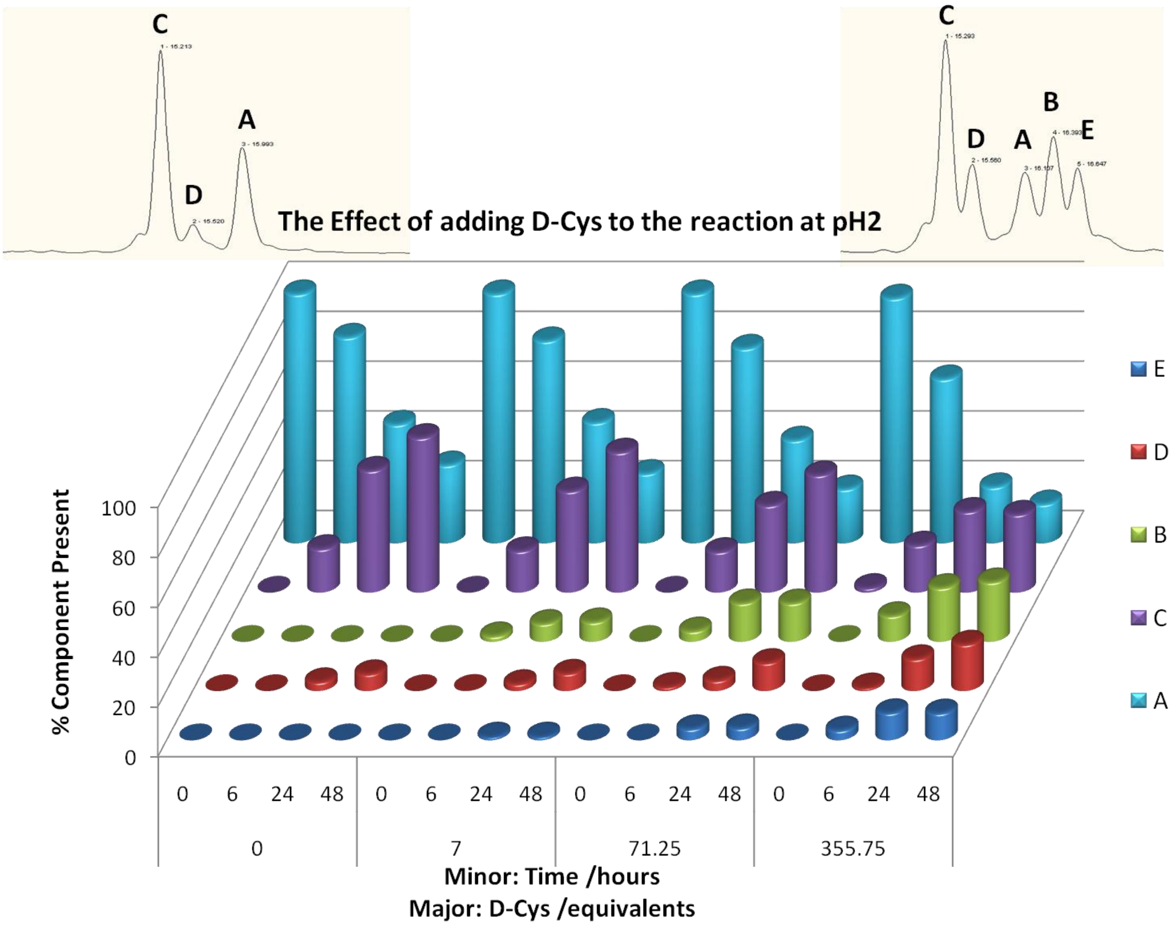

Supplement: Supplementary data [file mmc1.pdf]
